# Supplementary material for: How swarming bats can use the collective soundscape for obstacle avoidance
Source: PLoS Comput Biol. 2025 May 15;21(5):e1013013. doi: 10.1371/journal.pcbi.1013013 (PMC12180651; doi:10.1371/journal.pcbi.1013013)
Supplement: S1 File — (PDF) [file pcbi.1013013.s001.pdf]

Supplementary File S1

Statistic	Default Branched	No Others Branched	Default Curved	Default Widened	Perched 1 Branched	Perched 2 Branched	Differential Branched	Large Branched
**Median**								
Agent Dist. (md)	0.901	0.311	0.803	0.822	0.641	0.580	0.525	0.843
Wall Dist. (md)	1.583	2.292	1.419	1.682	2.087	2.131	2.163	2.188
Dur.	6.600	6.440	6.040	4.440	6.640	6.600	6.480	6.600
Steps	164	160	150	110	165	164	161	164
Agent $<$ 13 cm	0.036	4.850	0.000	0.000	0.177	0.143	0.183	0.055
Wall $<$ 13 cm	0.431	0.000	0.578	0.545	0.212	0.000	0.000	0.494
Wall Coll.	3	0	2	1	1	0	0	4
Wall Coll. (N), $\times$1000	0.727	0.000	0.533	0.364	0.242	0.000	0.000	0.488
Agents	25	25	25	25	25	25	25	50
**Min**								
Agent Dist. (md)	0.856	0.263	0.734	0.730	0.530	0.542	0.487	0.741
Wall Dist. (md)	1.406	2.276	1.231	1.548	2.065	2.079	2.136	1.778
Dur.	6.520	6.400	6.000	4.440	6.600	6.560	6.440	6.480
Steps	162	159	149	110	164	163	160	161
Agent $<$ 13 cm	0.000	2.384	0.000	0.000	0.000	0.000	0.000	0.000
Wall $<$ 13 cm	0.284	0.000	0.124	0.000	0.000	0.000	0.000	0.163
Wall Coll.	1	0	1	0	0	0	0	1
Wall Coll. (N), $\times$1000	0.244	0.000	0.267	0.000	0.000	0.000	0.000	0.123
Agents	25	25	25	25	25	25	25	50
**Max**								
Agent Dist. (md)	0.954	0.362	0.879	0.962	0.669	0.665	0.582	0.897
Wall Dist. (md)	1.950	2.303	1.932	1.787	2.175	2.178	2.211	2.540
Dur.	6.720	6.520	6.080	4.560	6.760	6.680	6.560	6.680
Steps	167	162	151	113	168	166	163	166
Agent $<$ 13 cm	0.252	9.522	0.164	0.205	0.565	0.422	0.435	0.215
Wall $<$ 13 cm	7.227	0.185	4.248	4.742	3.568	0.215	0.107	1.706
Wall Coll.	5	1	4	4	3	2	1	8
Wall Coll. (N), $\times$1000	1.220	0.252	1.067	1.455	0.723	0.491	0.245	0.994
Agents	25	25	25	25	25	25	25	50

Table A: Summary Statistics of For Different Conditions. See main text (Table 1) for details.

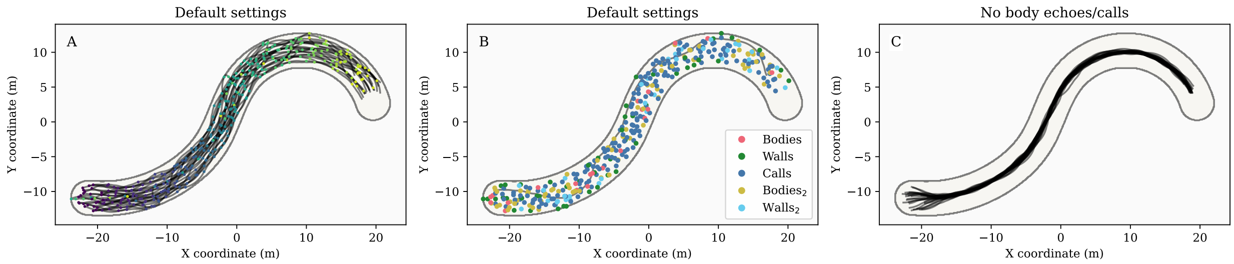

Fig A: Example paths of the paths taken by the agents in an arena that curves to the left and right. Similar to Fig 1.

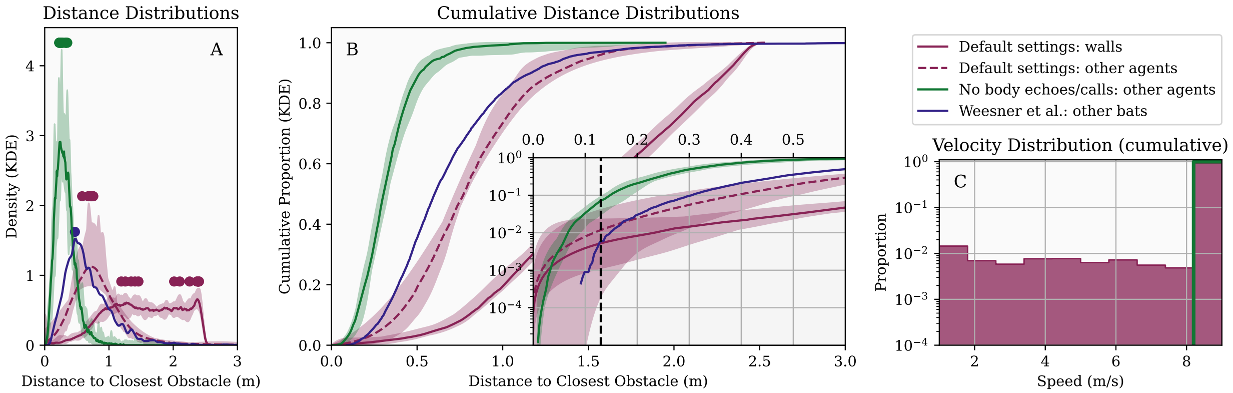

Fig B: Distributions of nearest distances for agents in the curved arena depicted in Fig A. Similar to Fig 3. The simulated distance distributions are plotted separately for each run to show the variation.

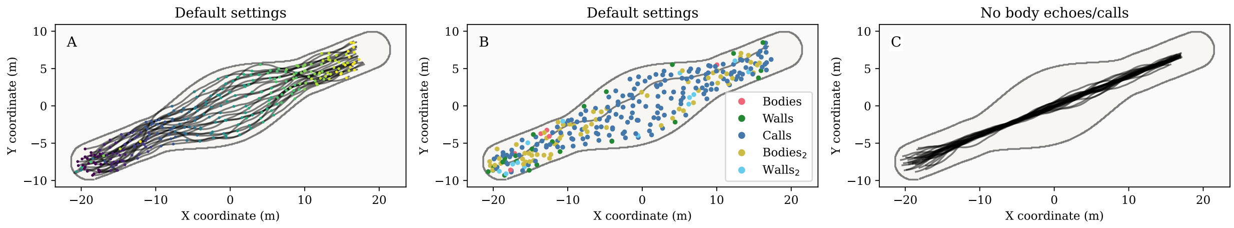

Fig C: Example paths of the paths taken by the agents in an arena that widens in the middle. Similar to Fig 1.

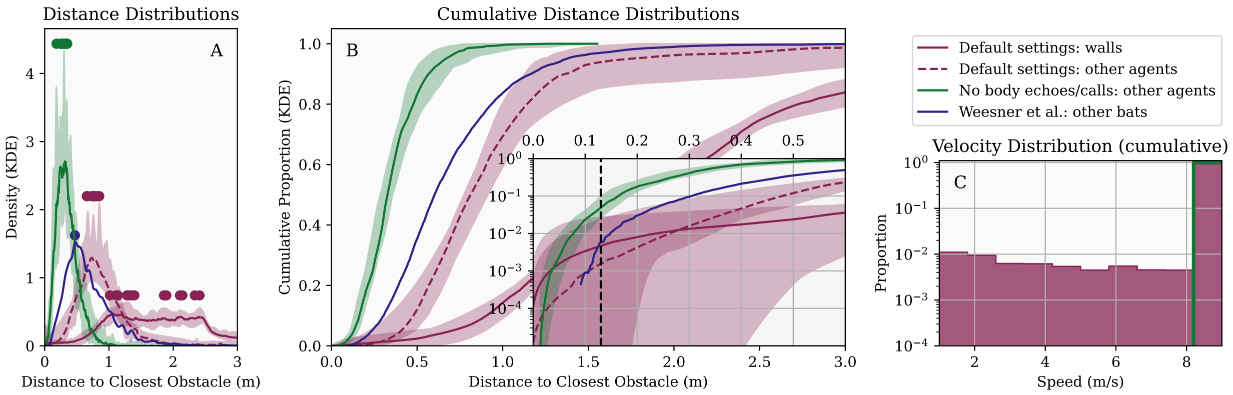

Fig D: Distributions of nearest distances for agents in the arena depicted in Fig C. Similar to Fig 3. The simulated distance distributions are plotted separately for each run to show the variation.

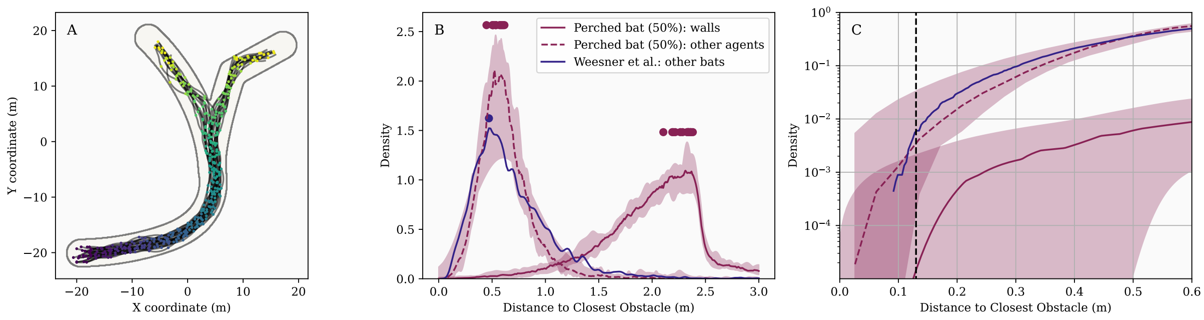

Fig E: Results for a variation of the simulation that included perched bats on the corridor walls (agents were placed at 50% of wall reflector locations). (A) Example of the paths taken by the bats (1 run out of the 15 iterations). (B) Distributions of the distances between agents compared with the interbat distances observed by Weesner et al. [4]. (C) The same data is used for panel (B) but visualized as cumulative distributions and focused on small distances. The simulated distance distributions are plotted separately for each run to show the variation.

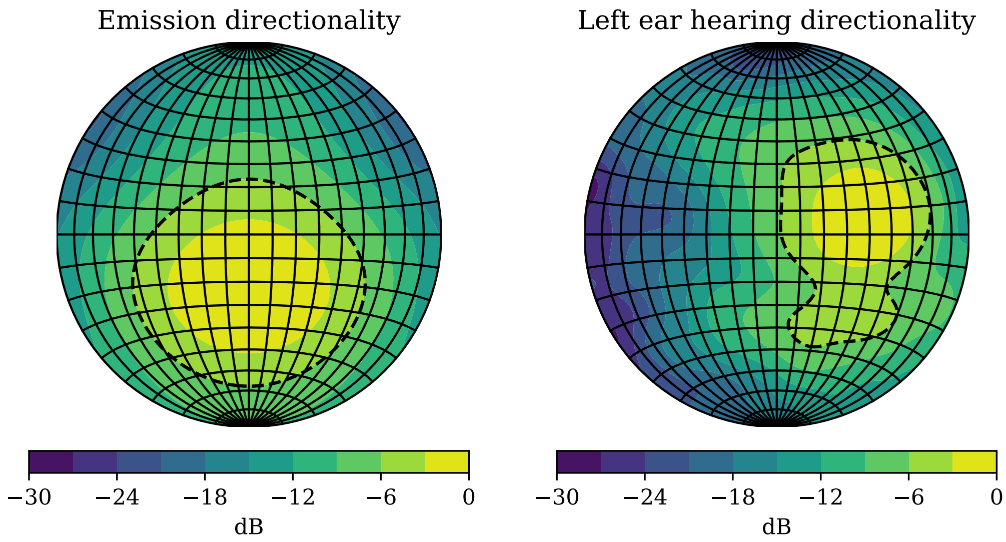

Fig F: Simulated directional patterns of *P. discolor* [1] used in this study (averaged across frequencies from 30 to 50 kHz). Left: emission beam directionality. Right: hearing directionality of the left ear (with the bat assumed to face the reader). The patterns are normalized to a maximum of 0 dB, with contours shown at 3 dB intervals. The -6 dB contour is represented by a dashed line to facilitate comparison with the findings of [2], who reported emission beam widths of six bats at half amplitude. The lines are spaced by 10 degrees.

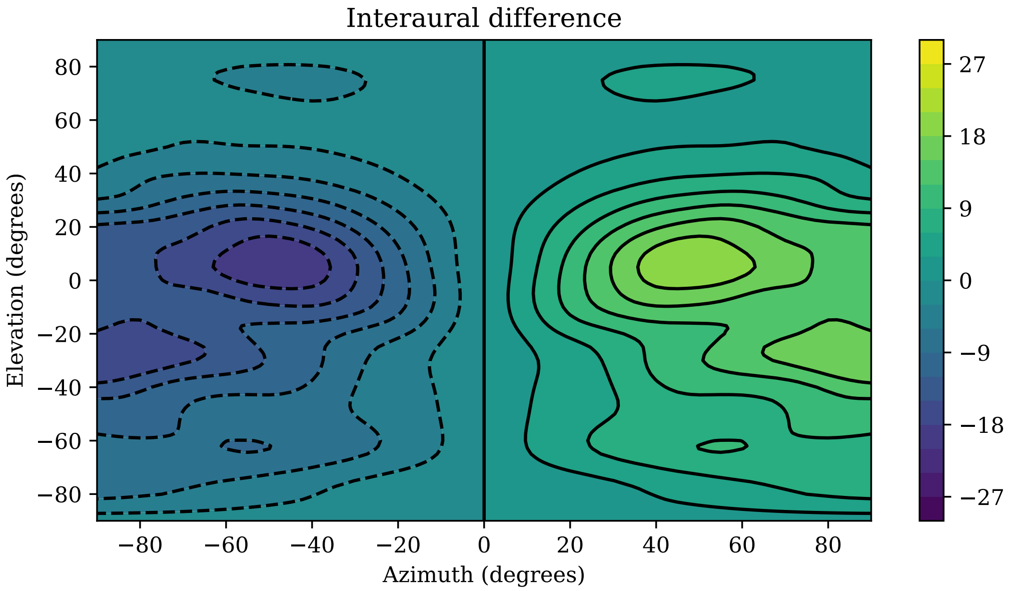

Fig G: Simulated Interaural Level Difference (ILD) as a function of azimuth and elevation for *P. discolor* [1] used in this study (averaged across frequencies from 30 to 50 kHz). The contour lines are spaced at 3 dB intervals. This representation is included to facilitate comparison with what is, to our knowledge, the only published hearing directionality data for *T. brasiliensis*. Obrist [3] reports ILD as a function of azimuth and frequency for *P. discolor* at an elevation of 0°. The current plot demonstrates that the hearing directionality used in this study is comparable to that of *T. brasiliensis*.

References

1. Vanderelst D, De Mey F, Peremans H, Geipel I, Kalko E, Firzlaff U. What noseleaves do for FM bats depends on their degree of sensorial specialization. PloS one. 2010;5(8):e11893.

2. Jakobsen L, Ratcliffe JM, Surlykke A. Convergent acoustic field of view in echolocating bats. *Nature*. 2013;493(7430):93–96.

3. Obrist MK, Fenton MB, Eger JL, Schlegel PA. What ears do for bats: a comparative study of pinna sound pressure transformation in Chiroptera. Journal of Experimental Biology. 1993;180(1):119–152.

4. Weesner A, Bentley I, Fullerton J, Kloepper L. Interaction rules guiding collective behaviour in echolocating bats. Anim Behav. 2023;206:91–98.
