# Supplementary material for: How swarming bats can use the collective soundscape for obstacle avoidance
Source: PLoS Comput Biol. 2025 May 15;21(5):e1013013. doi: 10.1371/journal.pcbi.1013013 (PMC12180651; doi:10.1371/journal.pcbi.1013013)
Supplement: Attachment — Submitted filename: BatSwarmRevision01.pdf [file pcbi.1013013.s002.pdf]

Reply to the reviewers

Data and Code Availability

We appreciate the reviewers bringing to our attention that some libraries were missing from the code we previously provided. In response, we have improved how we share our code, data, and associated files. Specifically, we have transitioned to using an Open Science Framework (OSF) repository, which offers greater flexibility compared to the previously used Zenodo platform. To ensure the code is fully functional, we created a clean copy of the repository to verify that all necessary files are included. This tested version has been directly uploaded to the OSF repository. Additionally, we generated a YAML file that lists the Python dependencies, enabling users to recreate the Anaconda environment we used for the simulations. We have also enhanced the documentation accompanying the code and data to provide a more detailed description of the files and their purpose.

The link to the OSF repository is `https://doi.org/10.17605/OSF.IO/QSM8C`.

Reviewer 1

1. The number of replicate simulations (runs) seems very small to me and there is no justification given for why 5 replicates per arena type is adequate. Similarly, the number of individuals in a run of the simulation (n=25) also seems very small compared to the numbers described earlier in the Introduction regarding the system which the simulations are representing. It would be good to see that the results are robust to higher numbers of individuals (if it is important to keep density constant, then the arena size can be scaled up appropriately). On a similar point, it would be good to see sensitivity analyses also for other chosen parameters such as the % in "we randomly placed the perched agents in 25% of the positions of the reflectors that make up the walls". [...] My guess would be that the simulations are very computationally expensive to run and the number of replicates is limited, but this does need acknowledging in the text.

The simulations are computationally demanding, which is why we chose to simulate fewer individuals in narrower passages to match the observed density in Tadarida brasiliensis. This is now explained in the paper. However, we recognize the importance of testing whether the mechanism scales to larger groups of individuals. To address this, we added a simulation with double the number of agents, scaling the horizontal (x and y) dimensions of the arenas by a factor of $\sqrt{2}$ to maintain a constant agent density. The results align closely with those from the simulations with 25 agents. While the total number of collisions increases by slightly less than a factor of 2, this is expected since the number of potential collisions rises with more agents (the collisions per bat decrease slightly).

2. More broadly, would the collective soundscape mechanism break down if different aspects of echolocation and hearing in the simulated bats was adjusted? This would allow the authors to test whether the calls and hearing have been tweaked by natural selection to optimize the collective soundscape effect; this wouldn't be possible (or ethical) with the real system, especially in the wild and with 1000s of bats, so a simulation approach would be well suited to test this.

To the best of our knowledge, the emission and hearing directionalities of Tadarida brasiliensis have not been measured in detail. The most relevant data available is limited in scope. Obrist et al. [1993] provides measurements of interaural hearing level differences as a function of az-

imuth and frequency, but this is insufficient to directly assess whether the emission or hearing directionality of Tadarida brasiliensis is optimized for the task modeled in our study. However, when comparing the interaural hearing level differences reported by Obrist et al. [1993] with those used in this paper, they appear to be very similar. Based on this, and in the absence of stronger conflicting evidence, we assume that the hearing directionality of Tadarida brasiliensis is neither unique nor specifically optimized for flying in large groups. In response to the reviewer's comment, we have now included figures illustrating the hearing, emission, and interaural level differences used in this study.

3. The first sentence of figure 2's legend needs to refer to the solid brown line. Is panel 2c referred to in the main text? Similarly, it wasn't clear how the inset in 2b helped.

We have updated the caption to make sure all lines are explicitly mentioned. We also made sure panel c is mentioned in the text. Regarding the inset, as we mention in the text, we think it helps to assess the difference in the distance distribution for smaller distances. Since the inset does not take up extra space in the paper or obscure other data, we opted for keeping it in the paper.

4. Figure 3's legend needs an overall title explaining what the figure shows.

We have added a title to the caption.

5. I didn't think that the choice of colors for the line graphs was the best for clarity (lines of similar colors, beige background) and I wondered how these would look printed black and white or to those with common forms of color blindness.

We appreciate the reviewer's concern regarding the clarity of our color choices, particularly for black-and-white printing and accessibility for individuals with color blindness. Our original colors were selected using the ColorBrewer tool, which is designed to offer colorblind-safe palettes. Nevertheless, to further enhance accessibility and ensure that the colors remain distinguishable in print, we have now adopted Paul Tol's color schemes for qualitative data, specifically the bright and muted sets (See `https://personal.sron.nl/~pault/data/colourschemes.pdf` for details). For sequential data, we have implemented the viridis colormap, known for its accessibility-friendly gradient. Additionally, we have adjusted the background color to a very light gray to improve contrast and clarity.

6. On page 10, references 10, 29 to support "akin to the mechanisms underlying murmurations in birds [e.g., 10, 29]" are not ideal, as murmurations have been directly studied in bird flocks – see the influential studies by Andrea Cavagna and colleagues. These would be much more relevant references to cite here.

We wish to thank the reviewer for pointing us to the work of Andrea Cavagna and colleagues, with which we were not familiar. Moreover, upon revisiting the references and their context, we agree with the reviewer that more directly relevant citations could have been used. In the new version of the paper, we now refer to two studies (one of which is by Cavagna and colleagues) that have empirically and theoretically assessed the role vision plays in mediating interactions and maintaining spacing in starling flocks. We agree with the reviewer that these are more relevant to the point that we wish to make, i.e., that bird flocks are assumed to use vision to maintain cohesion.

Reviewer 2

7. It is hypothesized that this helps avoid jamming and improves the selection of relevant echoes [reviewed by 27]." This statement is too brief to give full context of

the literature. Also, to my understanding, ref 27 actually posits an opposite view. Suggest adding the original references instead of citing a secondary reference.

We agree with the reviewer that ref 27 posits an opposite view. Nevertheless, this paper presents a relatively recent, comprehensive overview of the literature on jamming, contrasting findings in different species and under different conditions. That is why we referred this paper. However, we acknowledge the need to give a more detailed context. In response to the reviewer, we have now expanded this section with more references, including some more recent ones.

8. "A surprising alternative way" - it is important to consider that this paper specifically studies 2 bats at a time. The first question is if the 'going silent' mechanism really scales beyond a small group-size, which it may not. More importantly, considering these are pairs of bats trained to fly & likely housed together, it is also important to consider there may be social factors contributing to the silences that do not occur in the swarming contexts discussed just before (e.g. see Wright et al. 2014, Curr Biol). Would consider not including this sentence/citation.

We agree with the reviewer and have now removed this reference.

9. 'We created simulated arenas...' - what was the dimension of the arena. The density of points is mentioned, but not the actual size. While the actual size is mentioned later, it helps to put the 5m width here, and then refer to multiple variants modelled later.

We now mention the width of the corridors earlier in the text.

10. 'We did this to simplify the simulations, but also because bats following a flight path have been observed to maintain a constant flight height', also cite Kloerpper & Bentley 2017, Anim Behav - where they show stable flight heights during emergence.

We thank the reviewer for this suggestion and have now included this citation in the paper.

11. '...directionality of the bat Phillostomus discolor 's emission and hearing as simulated by Vanderelst et al. ' - this is an odd choice considering Phyllostomids are known to be particularly directional echolocators. This assumption will 'reduce' the intensity of the problem as the typical non-phyllostomid bat has an omnidirectional call emission.

We have now included a discussion of the hearing and emission pattern of *Phillostomus discolor* we used as compared with that of other bats. This discussion shows that the patterns we use are not more directional than those used by other bats. To the contrary, it seems that the patterns we used are slightly <u>less</u> directional than those reported for other bats, such as *Eptesicus fuscus* and other vespertilionid bats. To further allow the reader to assess this, we now also visualize the hearing and emission patterns in the supplementary material.

12. "This call rate also corresponds to the lowest rate observed by Lin et al. [25] for groups of Miniopterus fuliginosus leaving a cave" ...- though the methodology of cross-correlating a template call in reverberant audio makes one wonder about the actual call rates, which are much lower - which needs to be considered. Moreover, the likelihood of such a high call rate being sustained over a few seconds - a minute also seems low.

We agree with the reviewer regarding the pulse rates reported by Lin et al. Upon revisiting this reference, we observed that the authors explicitly acknowledge that their reported rates are likely influenced by wall reflections in the cave. Given this limitation, we have decided to no longer use this reference to support our selected call rate.

Unfortunately, apart from Lin et al., there is a lack of data on the echolocation and flight behavior of bats navigating caves. Specifically, there are no measurements available for how often Tadarida brasiliensis or any other bat species calls while inside caves, let alone while flying in groups. Similarly, the velocities of Tadarida brasiliensis inside caves seem to remain unmeasured. The only relevant information we could locate pertains to Tadarida brasiliensis entering caves, which shows that they decelerate as they approach the cave. They might reduce their velocity further upon entering.

In the absence of direct data on flight speed inside caves, we based our model on the speeds (~ 9 m/s) observed as Tadarida brasiliensis enter a cave, acknowledging that these bats decelerate during approach and may slow further once inside.

For the call rate, we now use data from Adams et al. [2017], who studied Tadarida brasiliensis flying in a flight room. These bats flew at speeds of 4–5 m/s (substantially slower than the speed we modeled), calling 15 to over 40 times per second depending on environmental complexity and social context. Higher call rates (up to 40 Hz) were achieved through call grouping, while in the presence of other (simulated or real) bats, the bats used an intermediate call rate of approximately 25 Hz. This intermediate rate reflects a balance between the maximum and minimum rates observed under different conditions and serves as a reasonable estimate for our model.

We now include the explanation above in the paper to highlight that we selected reasonable numbers for both flight speed and emission rate based on the limited data available in the literature.

13. Figure 1: 'b)The color of the dot shows which type of acoustic input was the loudest at each plotted position. We also plotted the path of a single selected agent.' - The line corresponds to the flight path one agent, while the dots correspond to the loudest sound type heard by the agent along the flight path. If this is the case, then how come the agent flying into the right-most flight path can hear loudest sounds that are from the left branch of the cave system?

It appears that our explanation of Figure 1b (and similar figures for different arenas and settings) was not sufficiently clear. In Figure 1b, the dots represent the positions (at 10-step intervals) of all simulated bats and indicate the strongest type of acoustic input received at these positions. The line, on the other hand, represents the trajectory of a single agent through space. Importantly, only the dots that fall on this trajectory line correspond to the inputs experienced by this specific agent. To address this potential confusion, we have updated the figure caption to emphasize that the dots do not all pertain to the single agent whose trajectory is shown.

14. Wrong hyperlinks - Fig. S1, S2 link to Main Figures 1 & 2!! I'm guessing the authors refer to the Figures in the Appendix?

We have fixed this technical issue.

15. 'As such, our results could be interpreted as expanding the results of Mazar and Yovel [27] by further reducing the challenge jamming poses to swarming bats' - this is really a semantic point. In some sense, the authors are not 'expanding', but actually shifting the paradigm of investigation here. 'Jamming' is a problem only if we consider a signal and a 'jammer'. The authors here have neatly shown that both are informative in some sense!

We agree with the reviewer. We have now added a few sentences to suggest this change in paradigm:

Our results build on the work of Mazar and Yovel [27] by not only demonstrating how the challenge of jamming is mitigated in swarming bats but also by shifting the perspective on 'jamming' itself. Rather than framing overlapping signals solely as a source of interference, our findings highlight that these signals can also carry useful information, reframing the paradigm from one of conflict to one of cooperative signal processing.

16. '..than the bats filmed by Weesner et al....' this 0.47 m matches the 0.5 m also reported by Theriault et al. 2010 ('reconstruction & analysis of 3d trajectories of Brazilian Free-tailed bats in flight') - which the authors may consider citing in earlier parts of the manuscript too.

We now refer to this work to justify the modelled flight speed. We also cite this paper when discussing the observed distances between bats.

17. Having read the methods, it seems like the simulations assume synchronised call emission from all agents at all times. Is this the case? If yes, then the authors need to make it explicit in the methods - and also discuss how this might actually make the task easier/harder for individual agents, vs. having unsynchronised call emissions. As shown in the cocktail party literature (and more specifically by Beleyur & Goerlitz 2019) unsynchronised calling across agents allows occasional auditory 'glimpses' of echoes too every now and then.

To clarify, our agents do not assume synchronized calls. As noted in Section 5.4, the timing of calls, secondary body echoes, and secondary wall echoes was randomized within the 30 ms interval for each simulation step to reflect unsynchronized call behavior. We agree with the reviewer that this is an important detail and have made this point more explicit earlier in the paper.

Regarding the discussion of Beleyur & Goerlitz (2019), we acknowledge that unsynchronized calling might result in occasional time windows where echoes arrive in isolation, potentially providing auditory "glimpses." However, we also note that Beleyur & Goerlitz (2019) do not propose a specific mechanism by which bats could reliably distinguish between self-generated echoes and those resulting from other calls or reflections in such scenarios. As such, we do not include this mechanism in our paper.

18. The call rate of 35 Hz seems very high esp for a bat that needs to fly for a few seconds to a minute maintaining that rate. The authors may consider discussing this choice. If a lower call rate had been used - perhaps the agents may have a harder time establishing the collective soundscape as their 'update rate' in come sense is lower?

Please see our response to the remark labelled as nr. 12. We have now lowered the emission rate. We also better justify the selected emission rate.

19. This may be my own misunderstanding of the methods - but it seemed like the agents take in the entire 30 ms audio and process it to generate their movement decisions. Whether biological echolocators take the entire interpulse-interval aside, this also inflates the amount of sensory information agents actually have access to. In contrast, when agents only consider the first X milliseconds (as the authors themselves have implemented in a previous paper) - this limits the amount of sensory information, and seems more realistic.

We appreciate the reviewer's comment. Similar to our previous implementations referenced by the reviewer, the current mechanism also reduces the binaural signals received by both ears to a single IID measure. The key difference is that, in this study, the measure is derived from the portion of the signal around its maximum value, whereas in our previous work, it was based on

the signal around the threshold crossing point. Therefore, the amount of sensory information available for motor control remains the same—one IID value.

That said, we agree that biological echolocators require time to process input. To address this, we now explicitly model separate phases for sensory data acquisition and processing/execution. Specifically, we assume that agents spend half the interpulse interval (20 ms, given a 25 Hz call rate) acquiring sensory input and the remaining 20 ms processing the data and executing motor commands. Thus, agents process only the acoustic input from the first 20 ms following call emission.

References

Amanda M. Adams, Kaylee Davis, and Michael Smotherman. Suppression of emission rates improves sonar performance by flying bats. Scientific Reports, 7(1):41641, January 2017. ISSN 2045-2322. doi: 10.1038/srep41641.

Martin K. Obrist, M. Brock Fenton, Judith L. Eger, and Peter A. Schlegel. What ears do for bats: A comparative study of pinna sound pressure transformation in chiroptera. Journal of Experimental Biology, 180(1):119–152, 07 1993. ISSN 0022-0949. doi: 10.1242/jeb.180.1.119. URL https://doi.org/10.1242/jeb.180.1.119.
